# CB1 Cannabinoid Receptor is a Target for Neuroprotection in Light Induced Retinal Degeneration

**DOI:** 10.3389/adar.2022.10734

**Published:** 2022-09-13

**Authors:** Manuel Soliño, Ignacio M. Larrayoz, Ester María López, Manuel Rey-Funes, Mariana Bareiro, Cesar Fabián Loidl, Elena Girardi, Laura Caltana, Alicia Brusco, Alfredo Martínez, Juan José López-Costa

**Affiliations:** ^1^ Instituto de Biología Celular y Neurociencia “Prof. E. De Robertis” (IBCN), Universidad de Buenos Aires-CONICET, Buenos Aires, Argentina; ^2^ Biomarkers and Molecular Signaling Group, Center for Biomedical Research of La Rioja (CIBIR), Logroño, Spain; ^3^ Angiogenesis Study Group, Center for Biomedical Research of La Rioja (CIBIR), Logroño, Spain

**Keywords:** cannabinoid, CB1, degeneration, neuroprotection, retina

## Abstract

In the last few years, an increasing interest in the neuroprotective effect of cannabinoids has taken place. The aim of the present work was to study the effects of modulating cannabinoid receptor 1 (CB1) in the context of light induced retinal degeneration (LIRD), using an animal model that resembles many characteristics of human age-related macular degeneration (AMD) and other degenerative diseases of the outer retina. Sprague Dawley rats (*n* = 28) were intravitreally injected in the right eye with either a CB1 agonist (ACEA), or an antagonist (AM251). Contralateral eyes were injected with respective vehicles as controls. Then, rats were subjected to continuous illumination (12,000 lux) for 24 h. Retinas from 28 animals were processed by GFAP-immunohistochemistry (IHC), TUNEL technique, Western blotting (WB), or qRT-PCR. ACEA-treated retinas showed a significantly lower number of apoptotic nuclei in the outer nuclear layer (ONL), lower levels of activated Caspase-3 by WB, and lower levels of glial reactivity by both GFAP-IHC and WB. qRT-PCR revealed that ACEA significantly decreased the expression of Bcl-2 and CYP1A1. Conversely, AM251-treated retinas showed a higher number of apoptotic nuclei in the ONL, higher levels of activated Caspase-3 by WB, and higher levels of glial reactivity as determined by GFAP-IHC and WB. AM251 increased the expression of Bcl-2, Bad, Bax, Aryl hydrocarbon Receptor (AhR), GFAP, and TNFα. In summary, the stimulation of the CB1 receptor, previous to the start of the pathogenic process, improved the survival of photoreceptors exposed to LIRD. The modulation of CB1 activity may be used as a neuroprotective strategy in retinal degeneration and deserves further studies.

## Introduction

Endocannabinoids (eCBs) are responsible for a wide range of physiological reactions that include modulation of synaptic function, control of tumor growth, analgesia, appetite stimulation, reduction of nausea, psychotropic effects, and synaptic plasticity, among others (1–4). The two most studied eCBs are N-arachidonoylethanolamide or anandamide (AEA) and 2-arachidonoylglycerol (2-AG). AEA is synthesized by phospholipase D (NAPE-PLD) from N-arachidonoyl phosphatidylethanolamine (NAPE) while 2-AG is synthesized by two diacylglycerol-lipase isoenzymes, DAGLα and DAGLβ. After binding to their receptors, eCBs are inactivated mainly by the fatty acid amide hydrolase enzyme (FAAH) and, to a lesser extent, by monoacylglycerol lipase (MGL), cyclooxygenase-2 (COX-2), and lipoxygenase (LOX) (5).

The effects of eCBs are mediated by metabotropic receptors (CB1 and CB2) and ionotropic receptor TRPV1. CB1 is the most abundant G-protein coupled receptor (GPCR) in the CNS. CB2 is also a GPCR that has been described in peripheral tissues, mainly in the immune system, but it has also been reported in the CNS including the retina (6–8). The activation of these receptors decreases the release of GABA and glutamate. TRPV1 is widely distributed in the CNS and, to a lesser extent, in the periphery. In addition, eCBs are ligands of peroxisome proliferator-activated receptor (PPAR-γ), which is involved in lipid metabolism, insulin sensitivity, regulation of inflammation and proliferation (9).

CB1 receptor and FAAH have been reported in the retina of rodents and primates (10, 11). High levels of CB1 receptor have been shown in the retina, iris and ciliary body of the human eye (12). The CB1 receptor and the degradative enzymes are mainly concentrated in the projection glutamatergic pathway. The CB2 receptor was reported in the retinal pigment epithelium (RPE), in photoreceptors, in the inner nuclear layer and in ganglion cells of the retina (13, 8). In the retina, cannabinoids regulate neurotransmitter release and modulate the retinal response to light. These results were supported by alterations of electroretinogram recordings in mice lacking CB1 and CB2 cannabinoid receptors (14).

Cannabinoid receptors protect CNS neurons from oxidative damage (15). In this regard, changes have been reported in eCB levels in diabetic retinopathy and age-related macular degeneration (AMD) (16). Cannabidiol significantly reduced both oxidative stress and neurotoxicity, and prevented retinal cell death in a rat model of diabetic retinopathy (17). Mixed agonists, which activate both CB1 and CB2, have a neuroprotective role in NMDA-induced excitotoxicity (18). Other studies have verified the protective role of the CB1 and TRPV receptors in glaucoma and retinal ischemia (19, 20). eCB agonists have being shown to exert a protective role in another model of glaucoma (21). The neuroprotective role of cannabinoids was also shown in an animal model of autosomal dominant retinitis pigmentosa (22).

Light induced retinal degeneration (LIRD) has been widely used as an animal model to study degenerative diseases of the retina (23–28). Continuous illumination (CI) produces photoreceptor degeneration, apoptosis in the outer nuclear layer (ONL), and synaptic degeneration in the outer plexiform layer (24, 29, 30, 31, 32, 33). The degenerative process starts in the outer retina, as happens in human AMD, juvenile macular degeneration, and retinitis pigmentosa (34). AMD is the first cause of acquired blindness in developed countries (35) and the majority of patients require indefinite treatment with antiangiogenic drugs or demonstrate disease progression despite therapies (36). This animal model is useful to study the potential neuroprotective effect of new drugs (37, 38). The aim of the present work was to study the effects of modulating the CB1 receptor in the LIRD model, in order to explore new therapies for AMD and other degenerative diseases of the outer retina.

## Materials and Methods

### Animals

Male Sprague Dawley albino rats (*n* = 28, body weight 200 g, age 60 days) were used. Animal care was performed in accordance with the Association for Research in Vision and Ophthalmology Statement for the Use of Animals in Ophthalmic and Vision Research. The animal model of continuous illumination and the experimental procedure was approved by the Institutional Committee for the Use and Care of Laboratory Animals of the Facultad de Medicina, UBA [CICUAL, Res. (CD) 3130/2017].

### Intravitreal Injections Protocol

Intravitreal injections were performed as previously described (37, 38). Animals were anestethized with Ketamine (40 mg/kg; Ketamina 50^®^, Holliday-Scott SA, Argentina) and Xylazine (5 mg/kg; Kensol^®^, König SA, Argentina). A drop of 2% lidocaine (Lidocaine^®^, Richmond SA, Argentina) was administered to each eye. Intravitreal injections (5 µl) were performed using a Hamilton syringe (Reno, NV, United States) and a 30-gauge needle. The right eyes received either ACEA (Sigma-Aldrich, Cat #A9719), a CB1 agonist, or AM251 (Sigma, Cat #A6226), a CB1 antagonist. The left eyes were used as controls (CTL) and received the same volume of vehicle (0.001% DMSO in 0.9 g/l NaCl). The final vitreal concentrations were 10 μM for ACEA and 2 μM for AM251. Doses were selected based on a previous scientific study (39).

### Continuous Illumination Procedure

After intravitreal injections and once animals recovered completely from anesthesia, rats were continuously illuminated for 24 h at 12,000 lux as previously described (37, 38). Illumination procedure was initiated during daylight period at 2 p.m. approximately. Groups of 3–5 rats were simultaneously placed in an open white acrylic box of 60 cm × 60 cm x 60 cm with 12 halogen lamps (12 V, 50 W each) located on top. Lighting level (12,000 lux) was determined using a digital illuminance meter. Temperature was maintained at 21°C and animals were offered food and water *ad libitum*. Immediately after completing the illumination protocol, 28 rats were sacrificed and their retinas were processed for qRT-PCR (*n* = 10), GFAP immunohistochemistry (IHC) and TUNEL technique (*n* = 8), or Western blotting (WB) (*n* = 10).

### Tissue Processing for Immunohistochemistry and TUNEL Assay

Rats were deeply anaesthetized by intraperitoneal injection of Ketamine and Xylazine as mentioned before and their eyes were removed. The cornea and lenses were cut off, and the remaining tissues were fixed by immersion in a solution containing 4% paraformaldehyde for 24 h. Eyes were embedded in paraffin and sectioned along a meridional plane in a Leica RM2125 RTA microtome (thickness: 5 µm). Prior to IHC or TUNEL, sections were subjected to an antigen retrieval protocol:Tris-EDTA Buffer (pH 9.0) at 90°C for 30 min.

### Immunoperoxidase Technique

Sections were incubated in methanol containing 3% hydrogen peroxide for 30 min in order to inhibit endogenous peroxidase activity. After washing in phosphate buffered saline (PBS), pH 7.4, sections were incubated in 10% normal goat serum for 1 h. Then, sections were incubated overnight with GFAP polyclonal primary antibody (Dako, Cat #Z0334, United States, dilution 1:500) at 4°C. The following day, sections were incubated sequentially in biotinylated goat anti-rabbit antibody (Sigma Chemical Co., MO; Cat #B8895, dilution 1:500) and in ExtrAvidin-Peroxidase^®^ complex (Sigma Chemical Co., MO., Cat #E2886, United States; dilution 1:500) at room temperature (RT) for 1 h. Development was performed using the DAB/nickel intensification procedure (40). Controls were performed by omitting primary antibodies (Supplementary Figure S1). Also retinas from non-illuminated and illuminated rats for 24 hs were stained by GFAP IHC as additional controls (Supplementary Figure S1).

### Terminal Deoxynucleotidyl Transferase dUTP Nick End Labeling Assay

Sections were processed using the ApopTag^®^ Peroxidase *In Situ* kit (Millipore, United States). Briefly, sections were washed in PBS and post-fixed in ethanol:acetic acid (2:1) at −20°C. After washing in PBS the endogenous peroxidase was inhibited as mentioned above. Then, sections were incubated sequentially with terminal deoxynucleotidyl transferase (1 h at 37°C) and with anti-digoxigenin conjugate (30 min at RT). Development was performed using the DAB/nickel intensification procedure followed by eosine counterstaining.

### Image Analysis of TUNEL and GFAP Immunoperoxidase Sections

Six retinal sections of both eyes from each experimental group were analyzed (ACEA, *n* = 4; AM251, *n* = 4). Anatomically matched areas of retina among animals were selected and. images were taken using a Zeiss Axiophot microscope attached to a video camera (Olympus Q5) under the same light conditions.

The following parameters were measured, blind to treatment, on 8 bits images, using the Fiji software (NIH, Research Services Branch, NIMH, Bethesda, MD):

*GFAP positive area*: Images of drug treated and control retinas were randomly selected. Immunoreactive area of the whole sections was thresholded. The region of interest (ROI) was the retinal surface between the two limiting membranes where Müller cells extend their processes. The GFAP positive area was calculated as the percentage of the ROI immunostained by GFAP.

*TUNEL positive nuclei/1000 µm* ^*2*^: Images of drug treated and control retinas were randomly selected and thresholded. As ROI, frames of 1000 μm^2^ were randomly determined on the ONL of treated and control retinas. The “analyse particles” function of Fiji was used and the TUNEL positive nuclei/1000 µm^2^ ratio was then obtained for each ROI.

### Western-Blotting

Retinas were homogenized (1:3, w/v) in lysis buffer (100 mM NaCl, 10 mM TrisHCL, 0.5% Triton X-100) plus 50 µl of Protease inhibitor cocktail (Merck KGaA, Darmstadt, Germany) at 4°C. Protein concentration was determined by the Bradford method. Then, 50–100 µl of each sample were mixed 4:1 with 5X sample buffer (10% SDS, 0315 M Tris-HCl, 25% beta-mercaptoethanol, 50% glycerol, 0.2 ml bromophenol blue 0.1%, pH 6.8), separated by 15% SDS–PAGE and transferred to polyvinylidenedifluoride membranes (GE healthcare life sciences, IL). Kaleidoscope Prestained Standards (Bio-Rad Laboratories, CA) were used as molecular weight markers. Membranes were blocked with PBS/5% non-fat dry milk and incubated overnight at 4°C with either a rabbit polyclonal antibody to GFAP (DAKO Inc., CA, United States; Cat #Z0334, dilution 1:500) or a rabbit polyclonal antibody to activated Caspase-3 (Sigma Chemical Co., MO, United States; Cat #H277, dilution 1:100) or a monoclonal anti-β-actin antibody (Sigma Chemical Co., MO, United States, Cat #C8487, dilution: 1: 1000). Membranes were incubated with ECL donkey anti-rabbit IgG, HRP-linked F (ab)2 fragment (Amersham), and were developed using a chemoluminiscence kit (SuperSignal West Pico Chemiluminescent Substrate, Thermo Scientific, MA). Membranes were exposed to X-ray blue films (Agfa Healthcare, Argentina), which were developed and then scanned with a HP Photosmart scanner (Hewlett Packard). Optical density was quantified by the Image Studio Light software of Li-Cor.

### RNA Isolation and Quantitative Reverse Transcription Polymerase Chain Reaction

The retinas of CTL and drug-treated retinas (ACEA and AM251, *n* = 5 per group) which were subjected to 24 h of continuous illumination were dissected out. Procedure was performed as detailed in Soliño et al. (37). Briefly, retinas were homogenized with TRIzol (Invitrogen, Madrid, Spain) and RNA was isolated with RNeasy Mini kit (Qiagen, Germantown, MD). Three µg of total RNA were treated with 0.5 µl DNAseI (Invitrogen) and reverse-transcribed into first-strand cDNA using random primers and the SuperScript III kit (Invitrogen).

Reverse transcriptase was omitted in control reactions. Resulting cDNA was mixed with SYBR Green PCR master mix (Invitrogen) for qRT-PCR using 0.3 µM forward and reverse oligonucleotide primers (Table 1). Quantitative measures were performed using a 7,300 Real Time PCR System (Applied Biosystems, Carlsbad, CA). Cycling conditions were an initial denaturation at 95°C for 10 min, followed by 40 cycles of 95°C for 15 s and 60°C for 1 min. At the end, a dissociation curve was implemented from 60 to 95°C to validate amplicon specificity. Gene expression was calculated using relative quantification by interpolation into a standard curve. All values were divided by the expression of the house keeping gene 18S.

**TABLE 1:** List of primers used in this study.

Gene	Primer orientation	**Primer sequence**
Bcl-2	Forward	CCG​GGA​GAA​CAG​GGT​ATG​ATA​A
	Reverse	CCC​ACT​CGT​AGC​CCC​TCT​G
Bax	Forward	AAA​CTG​GTG​CTC​AAG​GCC​CT
	Reverse	AGCAGCCGCTCACGGAG
Bad	Forward	GCC​CTA​GGC​TTG​AGG​AAG​TC
	Reverse	CAA​ACT​CTG​GGA​TCT​GGA​ACA
TNF-α	Forward	GAG​AGA​TTG​GCT​GCT​GGA​AC
	Reverse	TGG​AGA​CCA​TGA​TGA​CCG​TA
IL-1β	Forward	CCT​CTG​CCA​AGT​CAG​GTC​TC
	Reverse	GAA​TGT​GCC​ACG​GTT​TTC​TT
GFAP	Forward	GAA​GAA​AAC​CGC​ATC​ACC​AT
	Reverse	GGC​ACA​CCT​CAC​ATC​ACA​TC
iNOS	Forward	AGG​CCA​CCT​CGG​ATA​TCT​CT
	Reverse	GCT​TGT​CTC​TGG​GTC​CTC​TG
Ahr	Forward	TGA​TGC​CAA​AGG​GCA​GCT​TA
	Reverse	CAT​TGG​ACT​GGA​CCC​ACC​TC
CYP1A1	Forward	GGT​TAA​CCA​TGA​CCG​GGA​ACT
	Reverse	TGC​CCA​AAC​CAA​AGA​GAG​TGA
18S	Forward	ATG​CTC​TTA​GCT​GAG​TGT​CCC​G
	Reverse	ATT​CCT​AGC​TGC​GGT​ATC​CAG​G

### Statistical Analysis

The image data analysis of GFAP IHC and TUNEL studies of AEA-treated rats (*n* = 4) and AM251-treated rats (*n* = 4) were evaluated using D´Agostino, KS, Shapiro-Wilk, and F tests. In every case, Gaussian distribution was confirmed. Then, data were analysed using unpaired Student´s t-test (GraphPad Software, San Diego, CA). The results of WB (*n* = 5, per drug treatment), and qRT-PCR (*n* = 5, per drug treatment) were analysed using unpaired Student´s t-test. In every case, values are expressed as mean ± standard deviation. Differences were considered significant when *p* < 0.05.

The sample size was calculated based on data published by Soliño et al. (37). Free software (http://biomath.info/power/ttest.htm) was used to calculate the sample size. Power was set as 80% for an alpha of 5%, resulting in less than six animals per group to reach a significant improvement of the variable with an unpaired t-test.

## Results

### Effects of the Administration of ACEA on Light Induced Retinal Degeneration

#### Effects on Photoreceptor Apoptosis and Gliosis by Morphological Techniques

**FIGURE 1:**
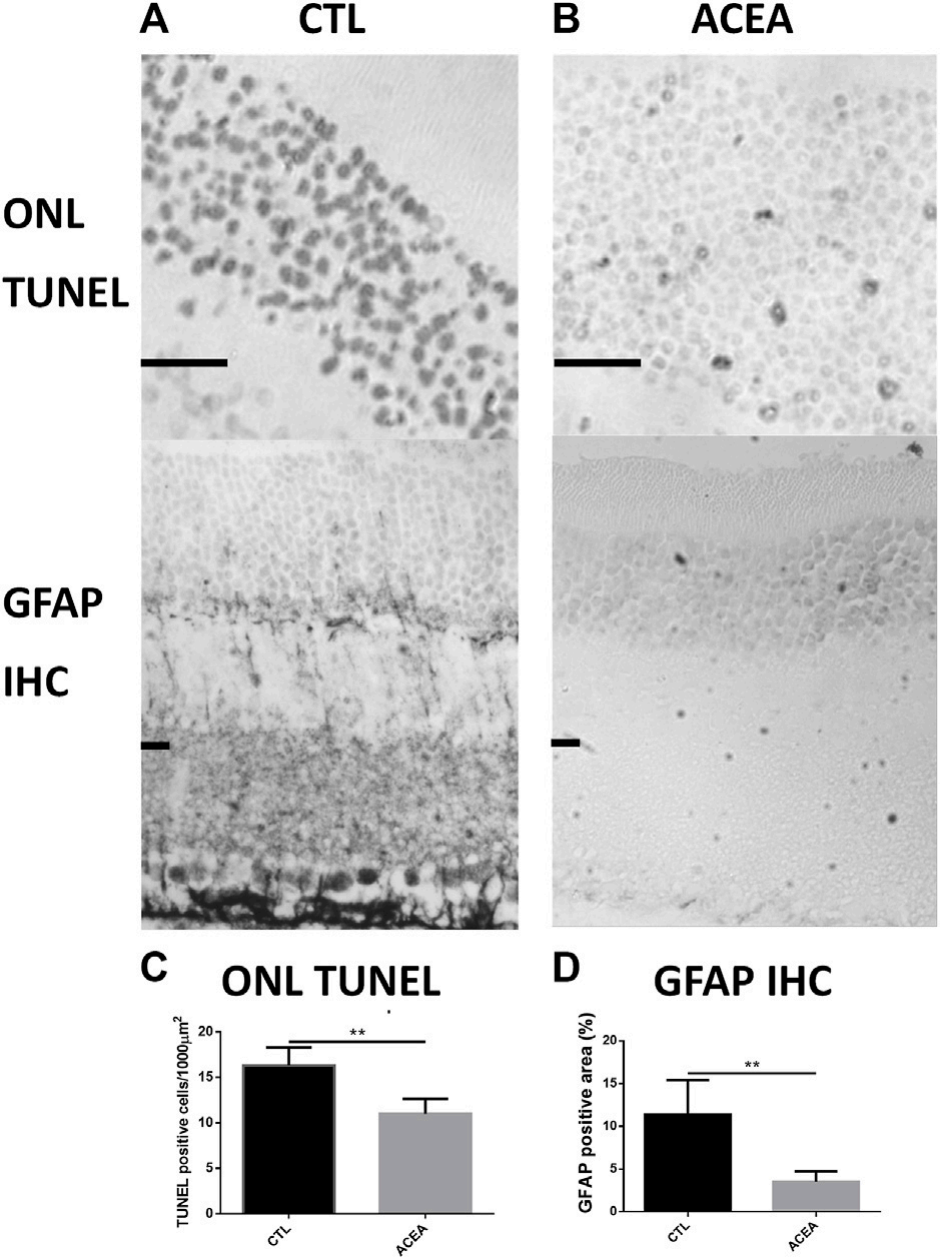
Treatment with ACEA decreased the number of apoptotic nuclei and GFAP-immunoreactive areas. **(A)**: Representative TUNEL stained sections of the outer nuclear layer (ONL) of the retina of a CTL eye (Left) and an ACEA-treated eye (Right). Observe the higher amount of apoptotic nuclei in the CTL eye compared to the ACEA-treated eye. Scale bar: 20 μm. **(B)**: Representative GFAP immunostained retinal sections of a CTL eye (Left) and an ACEA-treated eye (Right). A greater immunoreactivity of Müller cells is observed in the retina of the CTL eye compared to ACEA eye. **(C)**: Quantification of ONL TUNEL positive cells. **(D)** Quantification of positive GFAP immunostained area. **p < 0.01.

The quantification of apoptotic cells by TUNEL technique showed that treatment with ACEA decreased cell death. The eyes treated with ACEA had lower numbers of TUNEL positive nuclei in the ONL than the CTL eyes (11.01 ± 1.62 vs. 16.31 ± 1.98, *p* = 0.0041, *n* = 4) (Figure 1).

Quantification of the GFAP immunoreactive area in the ACEA-treated retinas showed a significant decrease of the glial reactivity compared to CTL (3.53% ± 1.19 vs 11.37% ± 4.06, *p* = 0.0181, *n* = 4) (Figure 1 and Supplementary Figure S1).

#### Effects on Apoptosis and Glial Reactivity by Western Blot

**FIGURE 2:**
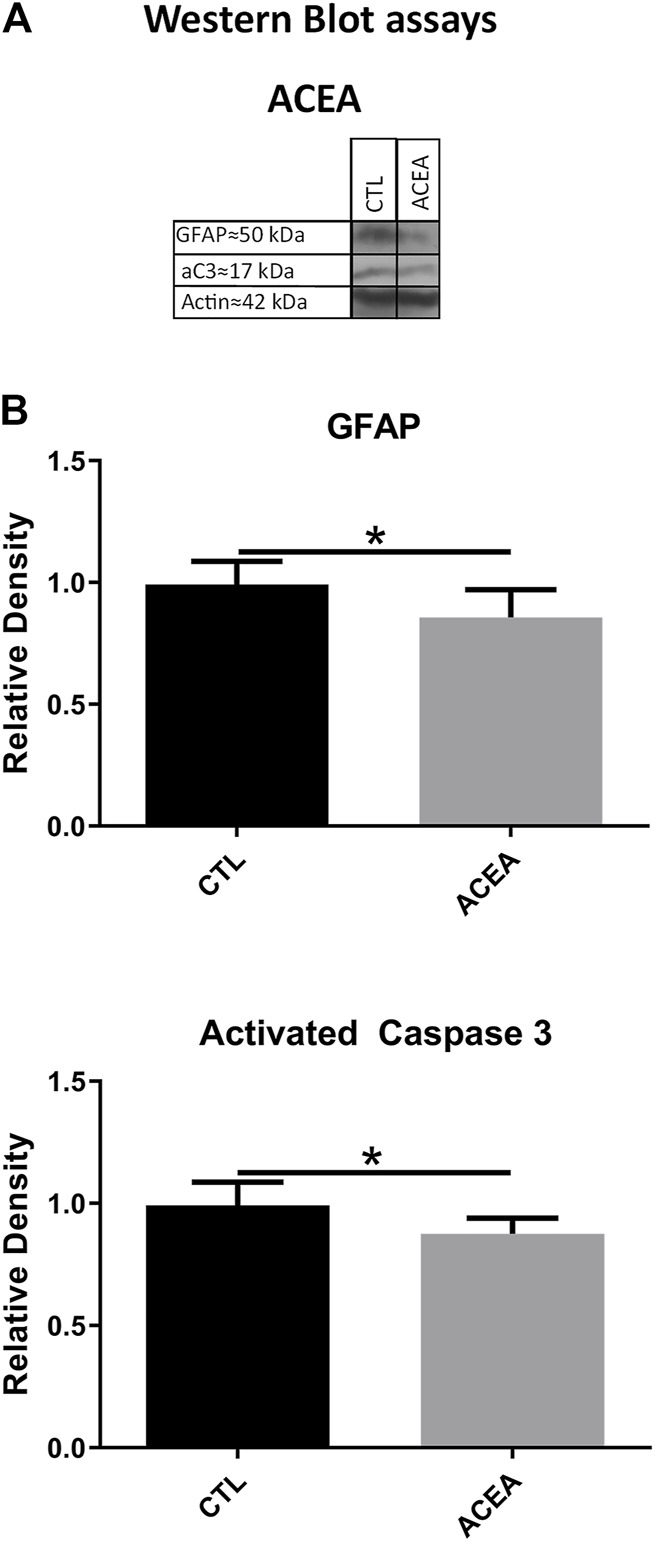
The treatment with ACEA decreased the levels of GFAP and activated Caspase-3 (aC3), **(A)**: WB of retinas from CTL and ACEA-treated eyes. **(B)** quantifications of GFAP and aC3 bands, Means and standard deviations are shown. *p < 0.05.

WB showed significant differences between the ACEA-treated eyes and their controls in the levels of activated Caspase-3 (aC3) and GFAP. ACEA decreased significantly the expression of aC3 (0.87 ± 0.06 vs 1.00 ± 0.09, *p* = 0.0329) and GFAP (0.85 ± 0.11 vs. 1.00 ± 0.09, *p* = 0.0484) (Figure 2).

#### Effects on Gene Expression (Quantitative Reverse Transcription-Polymerase Chain Reaction)

**FIGURE 3:**
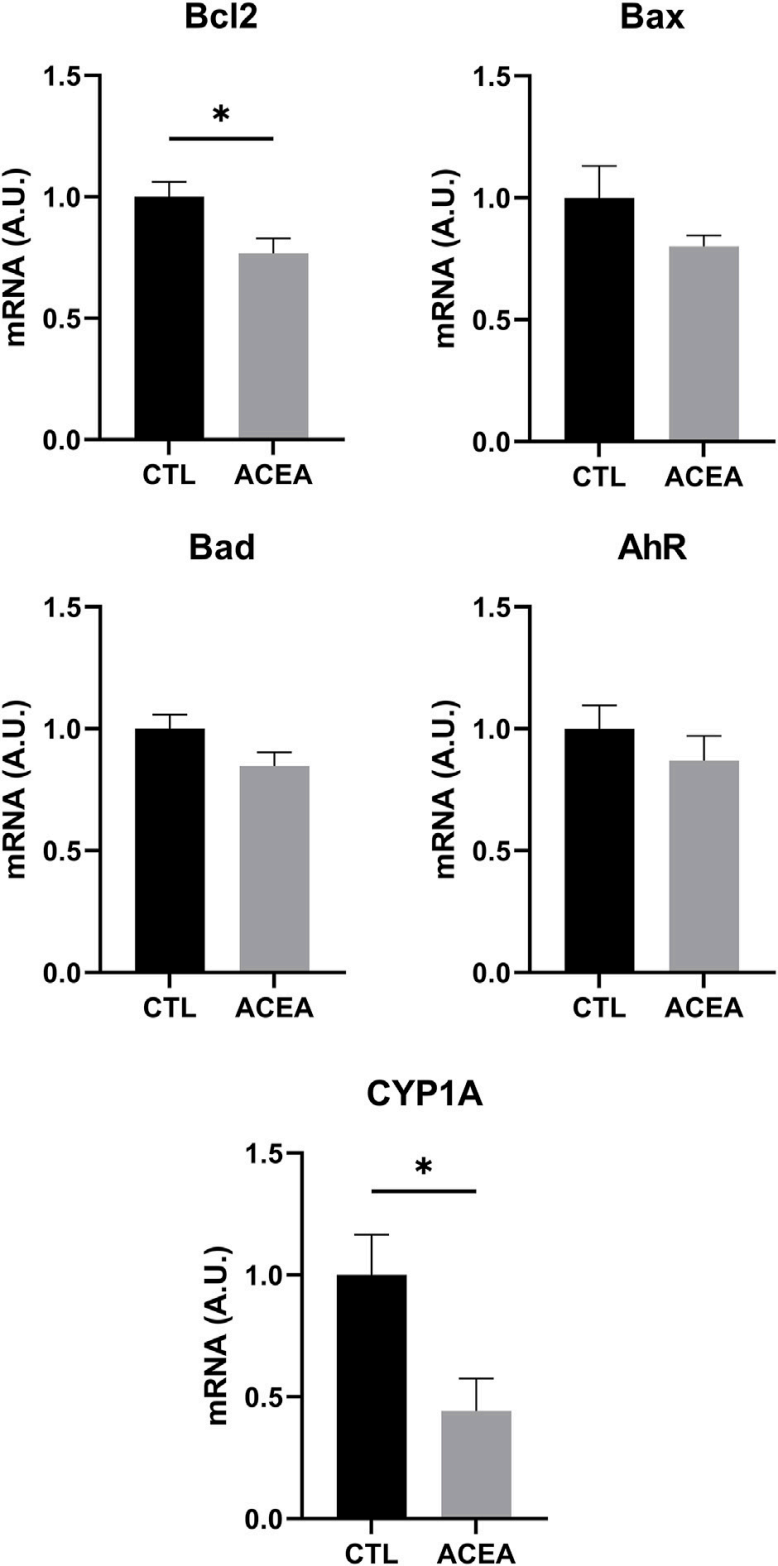
qRT-PCR of Bcl-2, Bax, Bad, AhR, and CYP1A1 of retinas from ACEA-treated eyes and their respective CTL. Means and standard deviations are shown. *p < 0.05.

**FIGURE 4:**
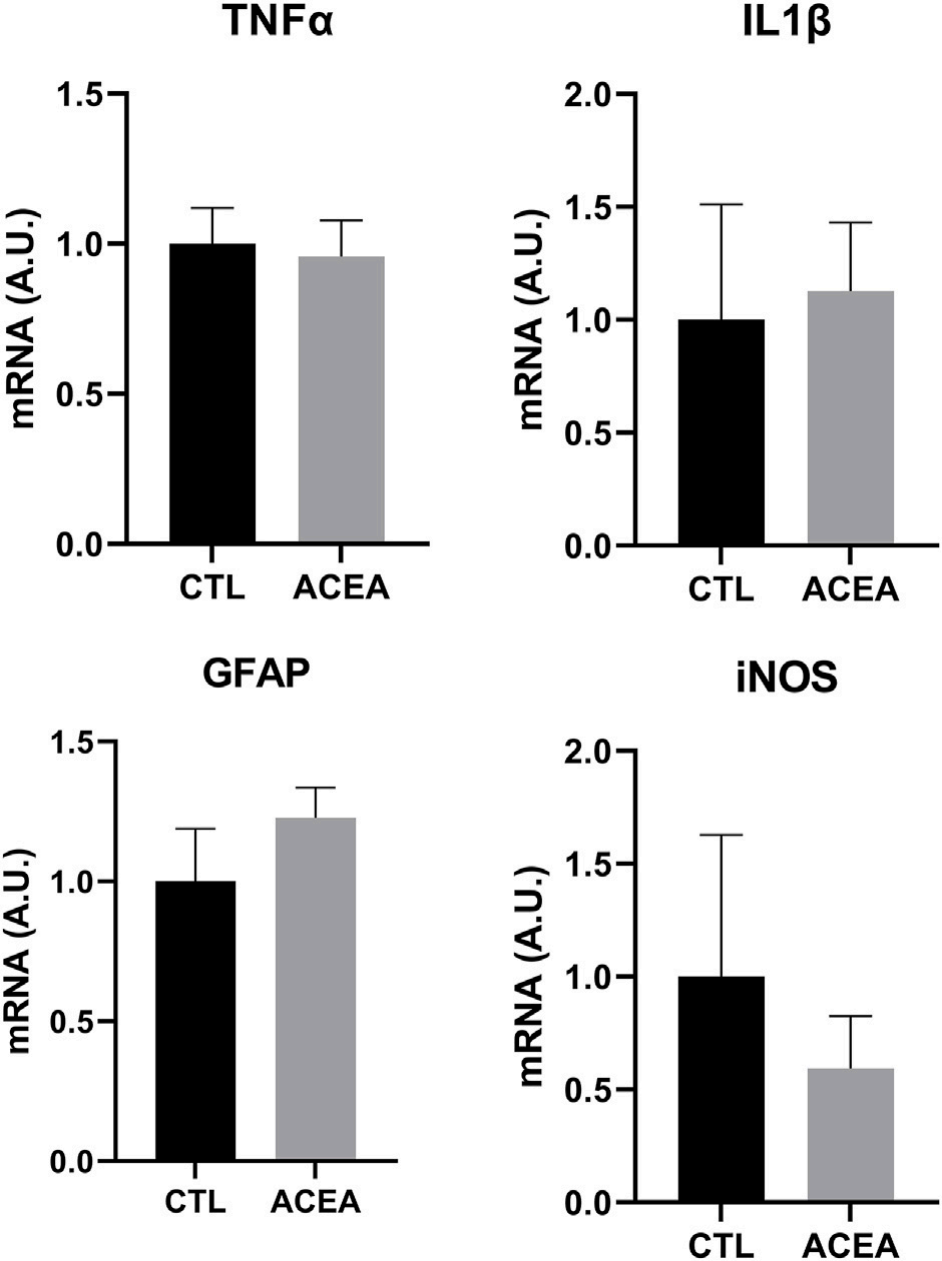
qRT-PCR of TNFα, IL1β, GFAP and iNOS from retinas of ACEA-treated eyes and their respective CTL. Means and standard deviations are shown.

In order to investigate the mechanisms involved in retinal protection, the expression of genes involved in apoptosis, inflammation, and cell response to xenobiotics were studied. We studied the effects of the modulation of CB1 on the transcription of the pro-apoptotic proteins Bad and Bax and the antiapoptotic Bcl-2. ACEA treatment decreased Bcl-2 expression (*p* = 0.0283) (Figure 3), but did not significantly change the expression of inflammatory markers IL-1β, TNFα, and iNOS. GFAP expression was not modified either (Figure 4).

The analysis of Aryl hydrocarbon Receptor (AhR), a transcription factor involved in the response to xenobiotics, and one of its target genes, CYP1A1, revealed that only CYP1A1was significantly decreased by ACEA treatment (*p* = 0.0307) (Figure 3).

### Effects of the Administration of AM251 on Light-Induced Retinal Degeneration

#### Effects on Photoreceptor Apoptosis and Gliosis by Morphological Techniques

**FIGURE 5:**
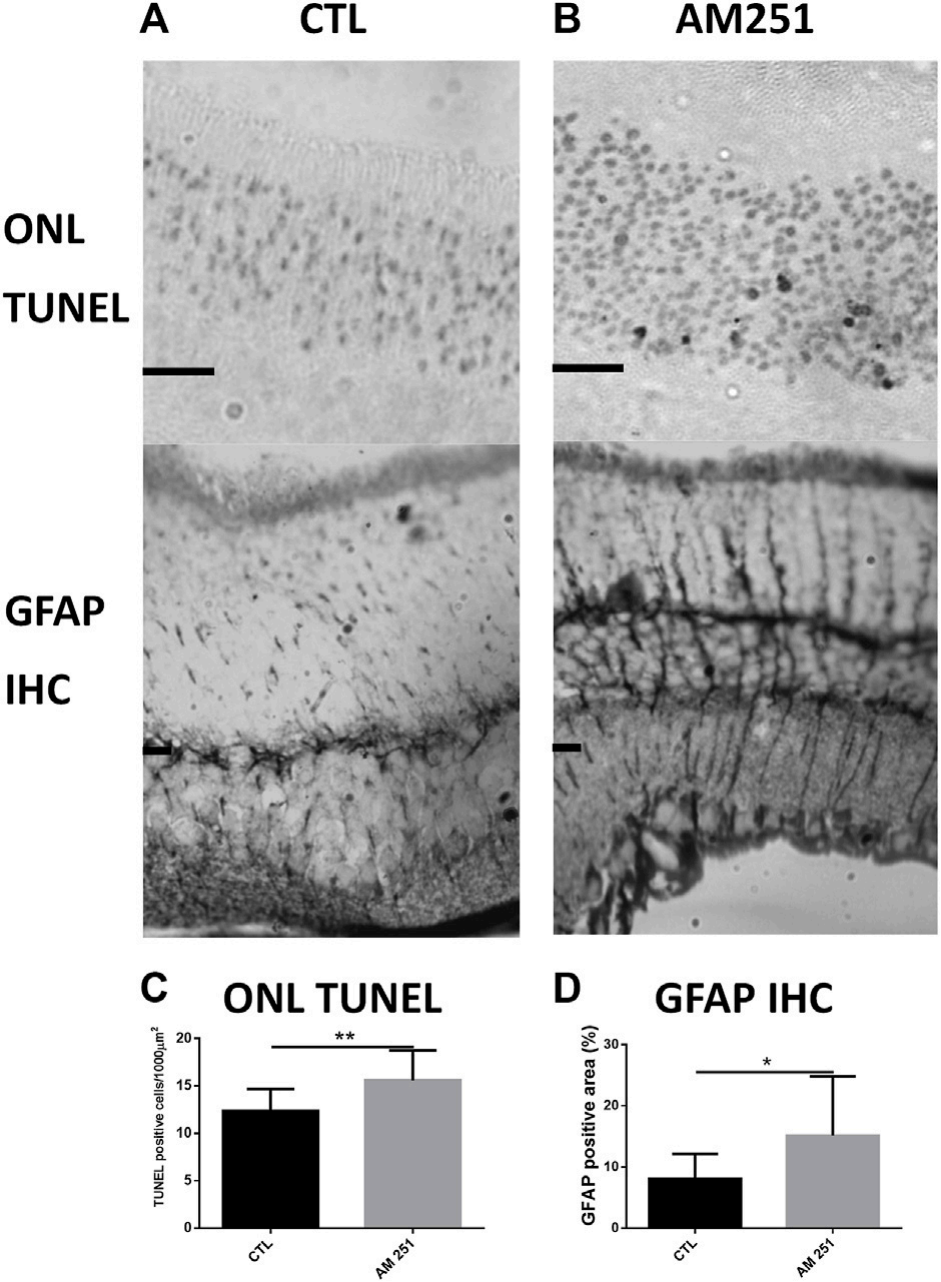
Treatment with AM251 increased the number of apoptotic nuclei and GFAP-immunoreactive areas. **(A)**: Representative TUNEL stained sections of the outer nuclear layer (ONL) of the retina of a CTL eye (Left) and an AM251-treated eye (Right). Observe the higher amount of apoptotic nuclei in the AM251-treated eye compared to the CTL eye. Scale bar: 20 μm. **(B)**: GFAP immunostained retinal sections of a CTL eye (Left) and an AM251-treated eye. A greater immunoreactivity of Müller cells is observed in the retina of the AM251-treated eye compared to the CTL eye. **(C)**: Quantification of ONL TUNEL positive cells. **(D)** Quantification of positive GFAP immunostained area. *p < 0.05; **p < 0.01.

The quantification of apoptotic cells by TUNEL in AM251-treated eyes showed a significant increase in cell death compared with CTL eyes. The eyes treated with AM251 presented higher densities of TUNEL positive nuclei in the ONL (15.61 ± 3.13 vs 12.36 ± 2.30, *p* = 0.0096) (Figure 5). Also an increase of the percentage of GFAP immunoreactive area was found in the eyes that received AM251 compared to CTL eyes (15.16% ± 9.67 vs 8.08% ± 4.10, *p* = 0.0207) (Figure 5 and Supplementary Figure S1).

#### Effects on Apoptosis and Glial Reactivity by Western Blot

**FIGURE 6:**
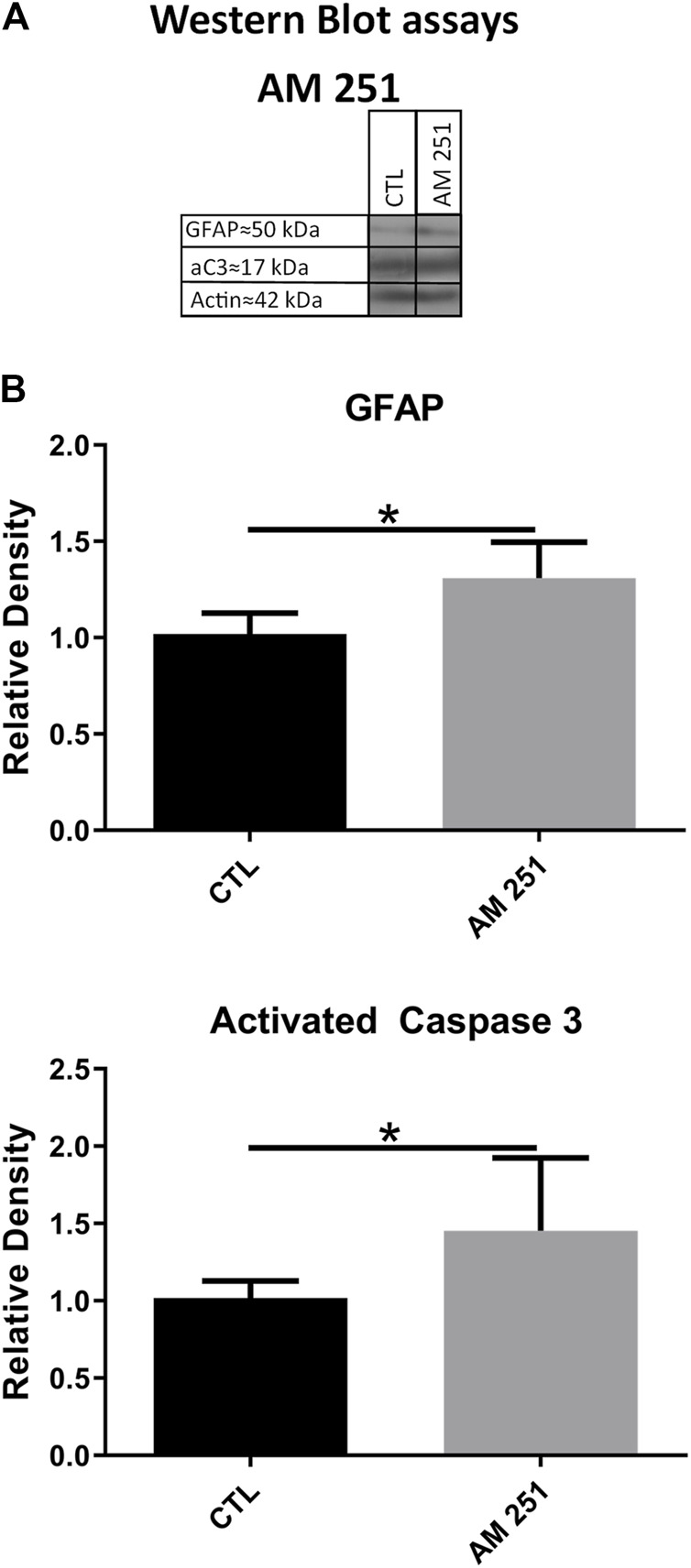
The treatment with AM251 increased the levels of GFAP and activated Caspase-3 (aC3). **(A)**: WB of retinas from CTL and AM251-treated eyes. **(B)**: Quantifications of GFAP and aC3 bands, Means and standard deviations are shown, *p < 0.05.

Intravitreal administration of AM 251 produced significant increases in the levels of GFAP (1.30 ± 0.18 vs. 1.00 ± 0.1, *p* = 0.0374) and aC3 (1.45 ± 0.47 vs 1.00 ± 0.10, *p* = 0.0441) when compared to the CTL eyes (Figure 6).

#### Effects on Gene Expression (Quantitative Reverse Transcription-Polymerase Chain Reaction)

**FIGURE 7:**
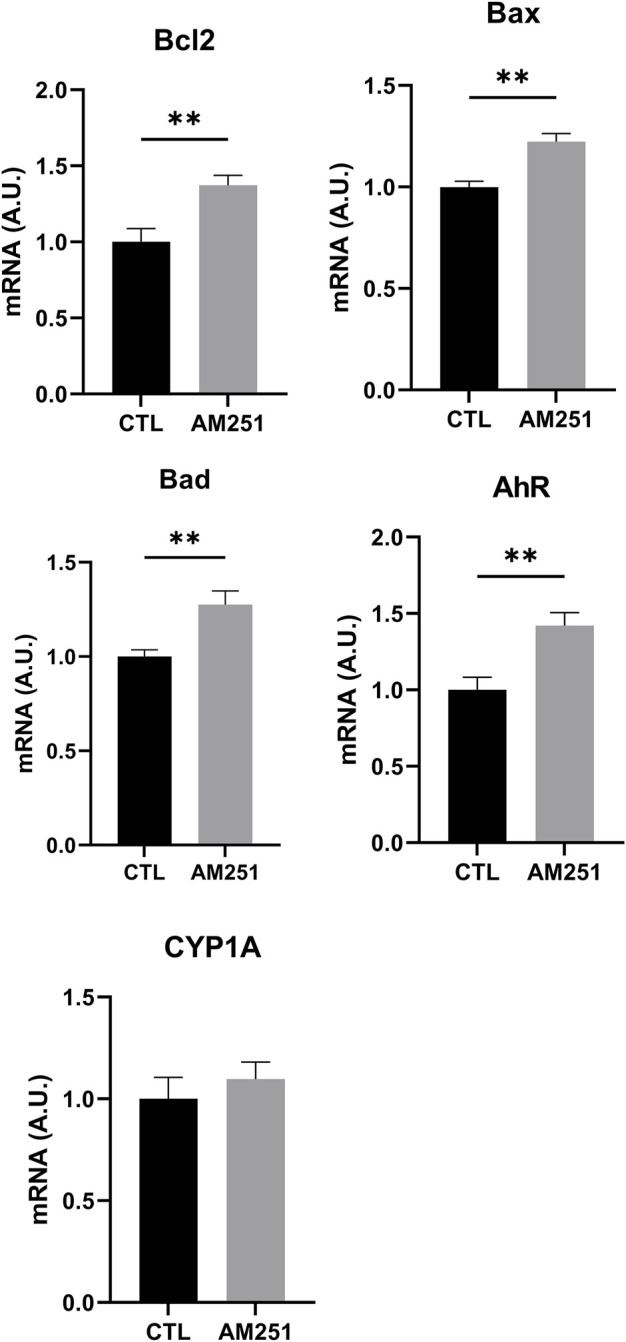
qRT-PCR of Bcl-2, Bax, Bad, AhR, and CYP1A1 of retinas from AM251-treated eyes and their respective CTL. Means and standard deviations are shown. **p < 0.01.

AM 251 increased the mRNA of the apoptotic proteins Bad and Bax (Bax, *p* = 0.002 and Bad, *p* = 0.0095), but it also increased the mRNA for Bcl-2 (*p* = 0.0096) (Figure 7).

**FIGURE 8:**
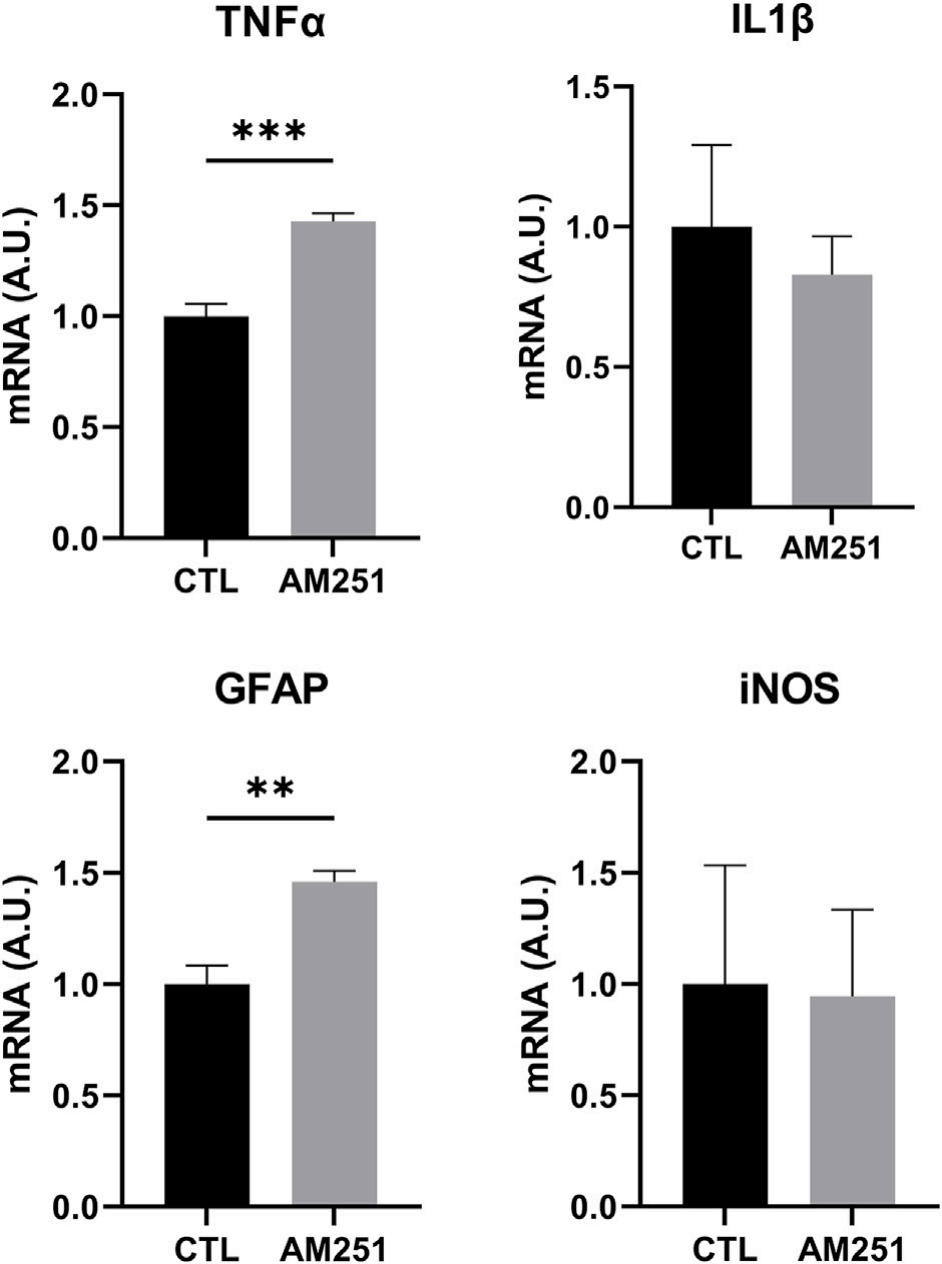
qRT-PCR of TNFα, IL1β, GFAP and iNOS from retinas of AM251-treated eyes and their respective CTL. Means and standard deviations are shown. **p < 0.01; ***p < 0.001.

When we studied the expression of inflammatory markers, AM251 increased significantly TNFα (*p* = 0.0002) but it did not change IL-1β and iNOS levels. AM 251 also increased significantly the mRNA levels of GFAP (*p* = 0.0014) (Figure 8).

In addition, AM251 increased significantly mRNA levels of AhR (*p* = 0.0074) (Figure 7).

## Discussion

In our model, the intravitreal injection of ACEA, a CB1 receptor agonist, prior to LIRD proved to be neuroprotective. Although intravitreal injections may not be practical for the treatment of human AMD sufferers, it is a valued experimental approach that enabled us to achieve a known concentration of the drug in the tissue avoiding problems of absorption and pharmacokinetics. However, this is a route of administration of VEGF antibodies for the treatment of wet AMD.

We observed a lower number of photoreceptor apoptotic nuclei in the ONL and we detected lower levels of activated Caspase 3 by WB. In addition, lower levels of glial reactivity were determined by GFAP IHC and WB. Conversely, the intravitreal injection of AM251, a CB1 antagonist, prior to CI proved to be deleterious. In this case, we found a higher number of apoptotic nuclei in the ONL and higher levels of activated Caspase 3 by WB. This was accompanied by higher levels of glial reactivity determined by GFAP IHC and WB.

The qRT-PCR results indicated that CB1 blockade by AM251 caused an increase in the mRNA of IL1β, GFAP and of the proapoptotic proteins Bax and Bad. Surprisingly, the mRNA of the antiapoptotic factor Bcl-2 increased with AM251 and decreased with ACEA. An alternative explanation to these results could be that protein levels do not follow mRNA levels of Bcl-2. In the proapoptotic condition, under AM251 treatment, free Bcl-2 levels decrease as it heterodimerizes with Bad and Bax and so the synthesis of more Bcl-2 mRNA is stimulated and the opposite could happen in the anti-apoptotic condition under ACEA treatment. It is known that continuous illumination induces free radical production (27) and probably the treatment with AM251 produced a more harmful environment as shown by an increased expression of AhR. This receptor is activated by xenobiotics but it also participates in development, differentiation, proliferation, immune response and apoptosis (41). Its activation triggers the expression of cytochrome P450 enzyme CYP1A1 and also the expression of suppressor cytokine signaling-2 (SOCS-2) which modulates inflammatory response (42, 43). Supporting this idea, we found that ACEA-treated retinas showed a lower transcription of CYP1A1, a gene regulated by AhR. Probably the neuroprotective effect of CB1 stimulation arises from the final balance of a set of signals (combinatory regulation).

Our results are in agreement with other reports that show the neuroprotective effect of the eCB system in CNS using different models of Alzheimer disease, excitotoxicity, ischemia, and oxidative stress. The neuroprotective effect of CB1 signaling was demonstrated by different approaches. For instance, the protective effect of WIN55212–2, a CB_1_/CB_2_ receptor agonist, was blocked by a CB_1_ receptor antagonist (44) and CB_1_ receptor-deficient mice showed larger infarct areas and lower blood flow in the ischemic penumbra (45). The protective mechanism of CB1 receptor may involve survival pathways such as inositol triphosphate (IP3) (46), PI3K (45), ERK (47), the reduction of the activation on the nuclear factor-κB (48), and the inhibition of Q-type Ca^2+^ currents or the activation of inward K^+^ currents (49).

The cumulative effect of light produces free radicals (50). Then, reactive oxygen species (ROS) peroxide membrane phospholipids, and polyunsaturated fatty acids (PUFA) that belong to outer PR segments, modify relevant proteins involved in signal transduction and induce DNA damage triggering apoptosis (51–53). Oxidative stress may also induce mitochondrial dysfunction that leads to neurotoxicity. This inability to adapt to the environmental stressor (light) could result in metabolic inflexibility that leads to neurodegeneration (54). In fact, severe ultrastructural alterations were detected in mitochondria in our model (24). Also, mitochondria are probably involved in triggering apoptosis by releasing cytochrome c induced by the increase of Bad and Bax whose expression was enhanced after the treatment with AM251.

In the retina, using an NMDA-induced excitotoxicity model, it was shown that the treatment with phytocannabinoids decreased the stress produced by reactive nitrogen species and lipid peroxidation, acting partially through CB1 (18). In a model of axotomy of the optic nerve, it was found that inhibition of FAAH improved the survival of ganglion cells by a CB1-dependent mechanism that decreased microglial reactivity (55). Another possible site of action of CB1 in the retina could be the microvasculature. In this regard, it was found that the diameter of the capillaries and the size of the pericytes of the blood-retinal barrier are regulated by CB1 in concert with nitric oxide (NO) and the activation of guanylate cyclase (56).

Opposite to our results, using a model of retinal degeneration induced by intraperitoneal administration of N-methyl-N-nitrosourea (NMU), the treatment with SR141716A (Rimonabant), a CB1 antagonist, induced the survival of photoreceptors, lowered the levels of glial reactivity and decreased vascular anomalies (57). Similar results were also obtained in a diabetic mouse model of retinal degeneration where the deletion of CB1 receptor or the use of SR141716 (Rimonabant) prevented retinal cell death (58). Finally, using a model of continuous illumination, saffron and selective CB1 and CB2 antagonists also reduced photoreceptor death in ONL and preserved visual function of the retina (59). These differences with our results may arise from alternative neuroprotective properties of SR141716A, not necessarily related with CB1 antagonism. It has been suggested that this compound could have inverse agonist properties and may function as agonist on other receptors (60). An alternative explanation is that at the dose of 10 μΜ employed in our study, ACEA could activate the CB2 receptor. If fact, CB2 receptor agonists have been shown to be neuroprotective in animal models of EAE and stroke (61). In our laboratory, the use of JWH133, a CB2 agonist, protected photoreceptors from cell death in the same LIRD model (unpublished data).

In conclusion, in our hands, the stimulation of CB1 improved the survival of photoreceptors and decreased glial reactivity in the LIRD model. The modulation of CB1 activity may be used as a therapeutic strategy in retinal degeneration either alone or in combination with accepted treatments and deserves further studies in other retinal degeneration models as well as in human retinal diseases.

## Data Availability

The original contributions presented in the study are included in the article/Supplementary Material, further inquiries can be directed to the corresponding author.
