# Supplementary material for: CB1 Cannabinoid Receptor is a Target for Neuroprotection in Light Induced Retinal Degeneration
Source: Adv Drug Alcohol Res. 2022 Sep 13;2:10734. doi: 10.3389/adar.2022.10734 (PMC10880786; doi:10.3389/adar.2022.10734)
Supplement: Supplementary file 1 [file Image1.pdf]

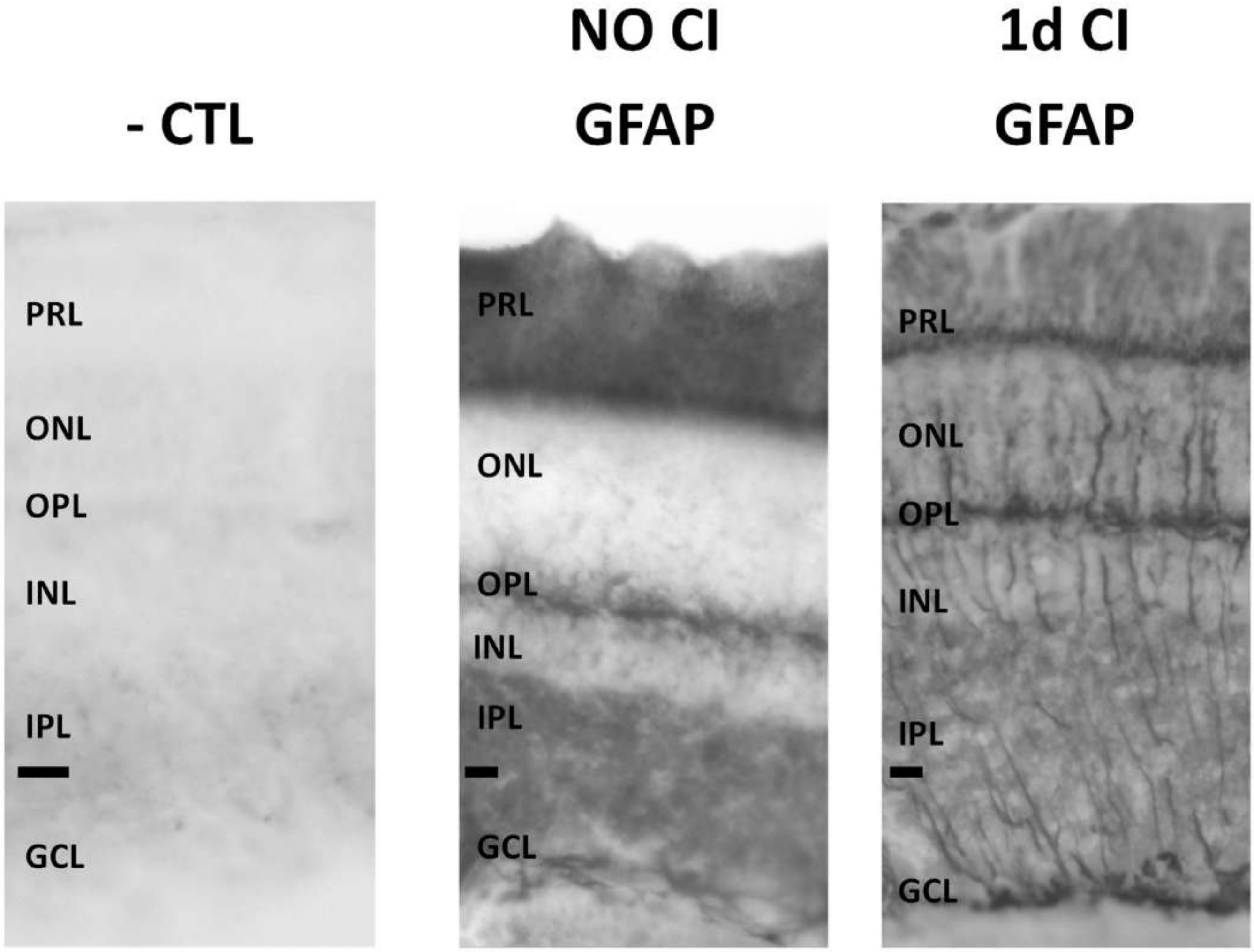

Left. CTL. Control retinal section from a rat illuminated for 1 day (24h). Negative control omitting primary antibody. Scale Bar= 10 µm. Middle. NO CI. Representative GFAP immunostained retinal section of a non-illuminated rat (NO CI). Scale Bar= 10 µm. Right. CI. Representative GFAP immunostained retinal section of a rat illuminated (CI) for 1 day (24 h). Scale Bar= 10 µm. PRL, photoreceptor layer; ONL, outer nuclear layer, OPL, outer plexiform layer; INL, inner nuclear layer; IPL, inner plexiform layer; GCL, ganglion cell layer.
